# Supplementary material for: Exploring the Therapeutic Potential of Ethyl 3-Hydroxybutyrate in Alleviating Skeletal Muscle Wasting in Cancer Cachexia
Source: Biomolecules. 2023 Aug 30;13(9):1330. doi: 10.3390/biom13091330 (PMC10527383; doi:10.3390/biom13091330)
Supplement: Supplementary file 1 [file biomolecules-13-01330-s001.zip › biomolecules-2551713-supplementary.pdf]

Supplementary Materials

Table S1. Aqueous metabolites identified from ^{1}H-NMR spectra recorded on aqueous metabolites derived from mouse gastrocnemius muscle.

Metabolite	δ^{1}H(ppm) and multiplicity	Moieties
Valine（Val）	0.99(d),1.05(d),2.26(m),3.60(d)	γ-CH_3, γ-CH_3, β-CH, α-CH
Isoleucine（Ile）	0.94(t),1.01(d),1.21(m),1.42(m),2.00(m),3.67(d)	δ-CH_3, γ-CH_3, half γ-CH_2, half γ-CH_2, β-CH, α-CH
2-Methylglutarate（2MG）	1.066(d),1.60(m),1.74(m),2.15(t)2.25(m)	C=O, C-OH, CH, CH_2, CH_3
Ethanol（Eth）	1.17(t),3.65(q)	δ-CH_3, CH_2
3-HB	1.20(d),2.30(q),2.39(q),4.14(m)	γ-CH_3, β-CH_2, γ-CH
Alanine（Ala）	1.47(d),3.78(q)	β-CH_3, α-CH
Leucine（Leu）	0.96(d),0.97(d),1.69(m),1.70(m),1.73(m),3.73(m)	α-CH_3, α-CH_3, γ-CH, β-CH_2, α-CH
Lysine（Lys）	1.43(m),1.50(m),1.73(m),1.89(m),1.92(m),3.02(t),3.75(t)	halfγ-CH_2, halfγ-CH_2, δ-CH_2,<br>β-CH_2, ε-CH_2, α-CH
Tyrosine（Tyr）	3.05(dd),3.19(dd),6.92(d),7.19(d)	halfβ-CH_2, halfβ-CH_2, β-CH, α-CH
Taurine（Tau）	3.24(t),3.41(t)	CH_2SO_3, NCH_2
Glycine（Gly）	3.57(s)	α-CH_2
Glucose（Glc）	β(3.24(dd),3.48(t),3.90(dd)), α(3.54(dd),3.71(t),3.72(dd),3.83(m))	β （H_2, H_3, H_5), α (H_2, H_3, H_6)
Lactate（Lac）	1.33(d),4.11(q)	β-CH_3, α-CH
Fumarate（Fum）	6.51(s)	CH
3-Methylhistidine（3-	3.09(m),3.20(m),3.71(s),3.93(m),7.04(s),7.	β-CH, β-CH’, CH_3, α-CH,

MH）	68(s)	H_2, H_4
Phenylalanine（Phe）	3.12(dd),3.30(dd),3.99(dd),7.33(d),7.37(t),7.43(t)	α-CH, half β-CH_2, half β-CH_2,<br>α-CH, β-CH, γ-CH
1-Methylhistidine（1-MH）	7.06(s),7.78(s)	CH (2), CH (4)
Anserine（Ans）	2.68(m),3.04(dd),3.21(m),3.78(s),4.49(dd),7.12(d),8.29(d)	CH (14), CH (7), CH (15), CH' (7),<br>CH (6), CH (8), CH (5), CH (2)
Carnosine（Car）	2.67(q),3.10(m),3.22(t),4.47(q),7.05(s),8.02(s)	CH_2, N-CH, CH, N-CH=N
Inosine（Ino）	3.83(d),3.84(d),6.1(d),8.23(s),8.35(s)	CH (2), N-CH=N, N-CH'=N
Fucose（Fuc）	1.22(d),1.26(d),3.45(dd),3.65(dd),3.78(m),3.86(dd),4.20(q),4.56(d),5.21(d)	CH, CH3, CH-O, O-CH-O
Proline（Pro）	1.99(m)	γ-CH_2
Glutamate（Glu）	2.08(m),2.12(m),2.34(m),2.37(m),3.75(m)	half β-CH2, half β-CH2, half γ-CH2, half γ-CH2, α-CH
Glutamine（Gln）	2.13(m),2.45(m),3.77(t)	γ-CH_2, β-CH_2, α-CH
Pyruvate（Pyr）	2.41(s)	α-CH_3
Glutathione（GSH）	2.15(m),2.55(m),2.96(m),3.77(m),4.56(m)	β-CH_2, γ-CH_2, CH_2-SH
Creatine（Cr）	3.04(s),3.93(s)	N-CH_3, α-CH_2
IMP	4.02(m),4.37(m),4.52(q),6.15(d),8.23(s),8.58(s)	CH_2, CH, CH', N-CH-O, N=CH-N. N=CH
ADP	6.13(d),8.27(s),8.58(s)	NH_2, δ-CH, CH (2)
ATP	6.14(d),8.27(s),8.58(s)	NH_2, δ-CH, CH (2)
Maltose（Mat）	3.27(q),3.42(t),3.57(dd),3.58(dd),3.59(m),3	CH (2), CH (10), CH' (2),

	.63(t),3.69(q),3.71(m),3.77(t),3.81(m),3.83 (m),3.93(m),3.96(t),4.66(d),5.24(d),5.41(d)	CH (8), CH (5), CH (9), CH (4), CH (11), CH (3), CH (12), CH (6), CH' (5), CH' (3), CH (1), CH' (1), CH (12)
UMP	3.40(m),4.26(m),4.35(m),4.41(m),5.99(m), 8.11(m)	N-CH, CH, CH', C-P, N=C-O, N-CH
NADP	6.05(d),6.15(d),8.16(s),8.42(s),8.83(d),9.11 (d), 9.29(s)	CH (32), CH (2), CH (12), CH (7), C (41), CH (43), CH (39)
Niacinamide（NA）	7.60(dd),8.23(dd),8.70(dd),8.92(s)	α-CH, β-CH, N=CH, N-CH

Table S2. Information of all metabolites in 1D ^{1}H-NMR spectra.

Metabolites	Mean ± SD			CAC vs.	CAC-K	CAC-K vs.	ANOVA	
	NOR	CAC	CAC-K	NOR	vs. CAC	NOR	F	P
Valine	1.269±0.168	2.530±1.286	1.112±0.331	↑*	↓*	ns	15.135	<0.001
Isoleucine	0.606±0.111	0.976±0.439	0.549±0.158	↑*	↓*	ns	10.179	<0.001
2-Methylglutarate	0.047±0.020	0.110±0.075	0.045±0.028	↑**	↓**	ns	8.475	<0.001
Ethanol	0.875±0.809	1.245±0.736	1.545±0.922	ns	ns	ns	2.334	0.111
3-HB	1.642±0.637	0.475±0.583	0.424±0.210	↓***	ns	↓***	27.005	<0.001
Alanine	12.075±1.245	12.678±2.614	13.270±1.943	ns	ns	ns	1.362	0.268
Leucine	2.646±0.420	5.055±2.178	2.690±0.640	↑*	↓*	ns	15.498	<0.001
Lysine	1.156±0.164	3.451±1.397	2.223±0.466	↑**	↓*	↑**	25.580	<0.001
Tyrosine	0.311±0.030	0.630±0.271	0.413±0.057	↑**	ns	↑**	14.915	<0.001
Taurine	139.670±4.964	140.164±12.16	146.6±15.504	ns	ns	ns	1.517	0.232
Glycine	7.908±1.189	7.540±1.552	10.665±2.882	ns	↑**	↑*	9.391	<0.001
Glucose	3.758±0.468	2.341±2.342	5.010±1.430	ns	↑*	↑*	10.101	<0.001
Lactate	52.016±2.791	33.496±26.359	63.901±10.710	ns	↑*	↑**	13.215	<0.001
Fumarate	0.280±0.058	0.130±0.039	0.232±0.065	↓***	↑***	ns	20.811	<0.001
3-Methylhistidine	5.620±0.360	6.815±2.376	4.901±0.653	ns	ns	ns	7.176	0.002
Phenylalanine	0.329±0.030	0.555±0.159	0.409±0.119	↑**	↓*	↑*	12.006	<0.001
1-Methylhisdine	0.362±0.209	0.212±0.068	0.205±0.261	ns	ns	ns	2.434	0.101
Anserine	10.229±0.657	10.343±1.120	9.174±1.131	ns	↓*	↓*	6.113	0.005
Carnosine	2.636±0.198	2.031±2.354	2.349±0.609	ns	ns	ns	0.670	0.518
Inosine	0.408±0.080	0.718±0.290	0.499±0.100	↑*	ns	↑*	10.519	<0.001
Fucose	1.295±0.072	0.884±0.626	1.566±0.254	ns	↑*	↑*	11.750	<0.001
Proline	1.465±0.342	2.271±0.934	1.314±0.324	↑*	↓*	ns	10.553	<0.001
Glutamine	0.526±0.202	0.157±0.126	0.418±0.535	↓***	ns	ns	3.089	0.057
Glutathione	1.572±0.262	0.949±0.356	1.436±0.191	↓***	↑**	ns	17.991	<0.001
Creatine	157.157±6.808	160.192±17.961	166.120±20.959	ns	ns	ns	1.099	0.343
IMP	20.386±0.984	19.575±2.574	21.046±2.729	ns	ns	ns	1.399	0.259
ADP	0.540±0.040	0.545±0.059	0.361±0.058	ns	↓***	↓***	57.919	<0.001
ATP	0.199±0.036	0.126±0.069	0.166±0.029	↓*	ns	↓*	7.789	0.001
Maltose	0.784±0.134	0.562±0.596	0.886±0.526	ns	ns	ns	1.644	0.207
UMP	0.220±0.016	0.211±0.062	0.240±0.038	ns	ns	ns	1.769	0.184
NADP	0.100±0.017	0.089±0.028	0.074±0.025	ns	ns	ns	4.297	0.021
Niacinamide	0.565±0.044	0.526±0.068	0.582±0.096	ns	ns	↓*	1.880	0.167
Glutamate	3.465±0.679	3.317±0.790	2.593±0.565	ns	↓*	↓**	7.390	0.002
Pyruvate	0.539±0.208	0.166±0.134	0.445±0.546	↓***	ns	ns	3.079	0.058

* Statistical significances：ns: no statistical significance, $p > 0.05$; *：$p < 0.05$; **：$p < 0.01$; ***：$p < 0.001$, Red represents up-regulation, while blue for down-regulation.

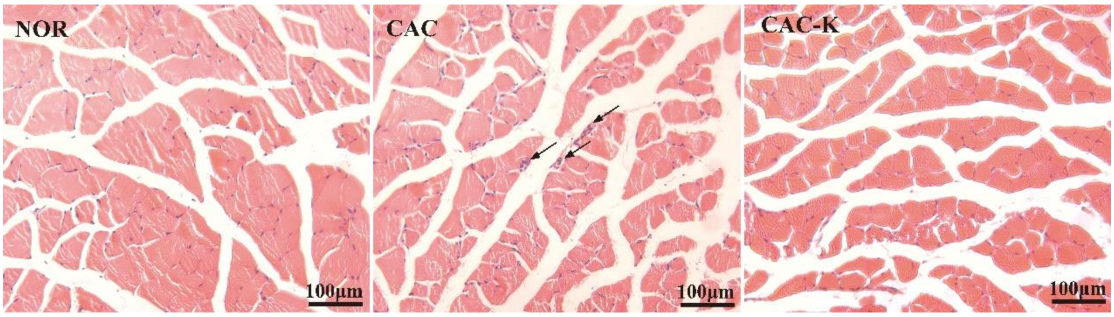

Figure S1. EHB administration improved infiltration of inflammatory cells in the cachectic gastrocnemius muscle of colon cancer cachexia mice. Hematoxylin-eosin staining in gastrocnemius muscle. Arrows indicate inflammatory cell infiltration.

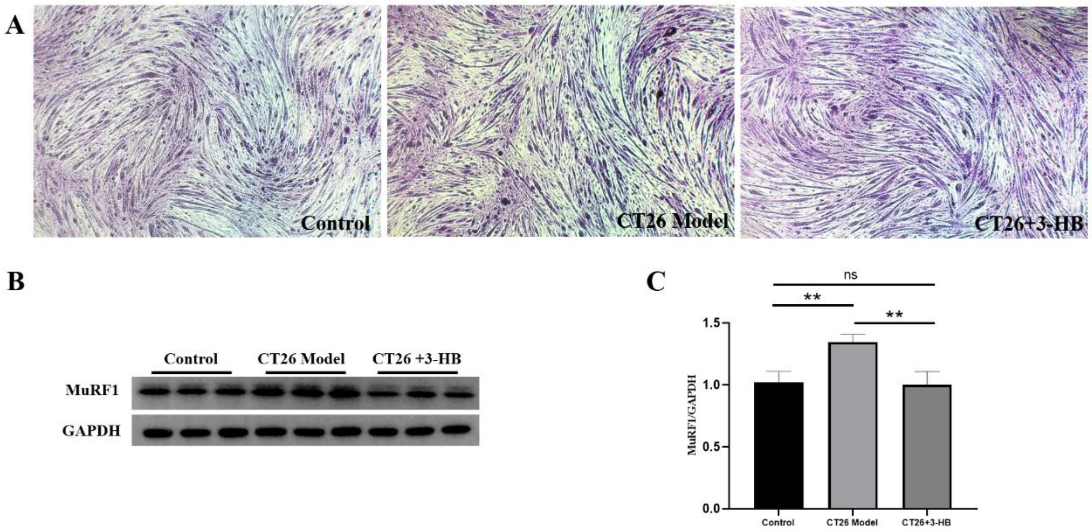

Figure s2. 3-HB supplementation alleviated C2C12 myotube atrophy induced by the culture supernatant of CT26 cell lines. (A) Morphological images of normal control myotubes (the Control group), myotubes treated with CT26 culture supernatant (the CT26 Model group), myotubes treated with CT26 culture supernatant and 3-HB (the CT26+3-HB group). (B) Western blot analysis of MuRF1 expression in Control, CT26 Model and CT26+3-HB groups of myotubes (n=3 for each group). (C) Quantitative comparisons of MuRF1 expressions between these three groups. Statistical significances: $p > 0.05$, ns; $p < 0.01$, **.

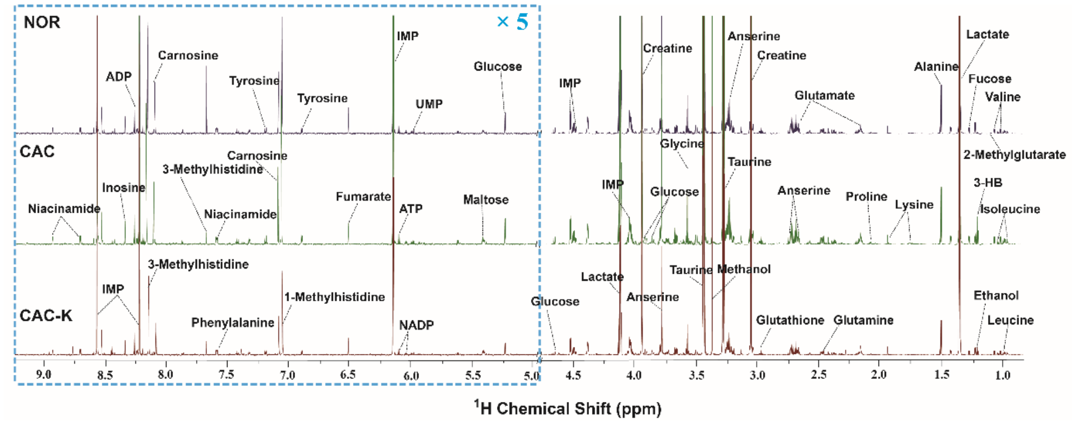

Figure S3. Representative 1D ^{1}H-NMR spectra recorded on aqueous metabolites extracted from the three groups of mouse gastrocnemius. The NMR experiments were performed at 25 °C with a Bruker Avance III 850 MHz spectrometer. Spectral regions of 0.75-4.75 ppm and 4.95-9.5 ppm are displayed, while the water region of 4.75-4.85 ppm was removed. The region of 4.95-9.5 ppm has been magnified 5 times for the purpose of clarity. Abbreviations: 3-HB, 3-Hydroxybutyric acid; ATP, adenosine triphosphate; ADP, adenine diphosphate; IMP, inosine monophosphate; UMP, uridine monophosphate; NADP, nicotinamide adenine dinucleotide phosphate.

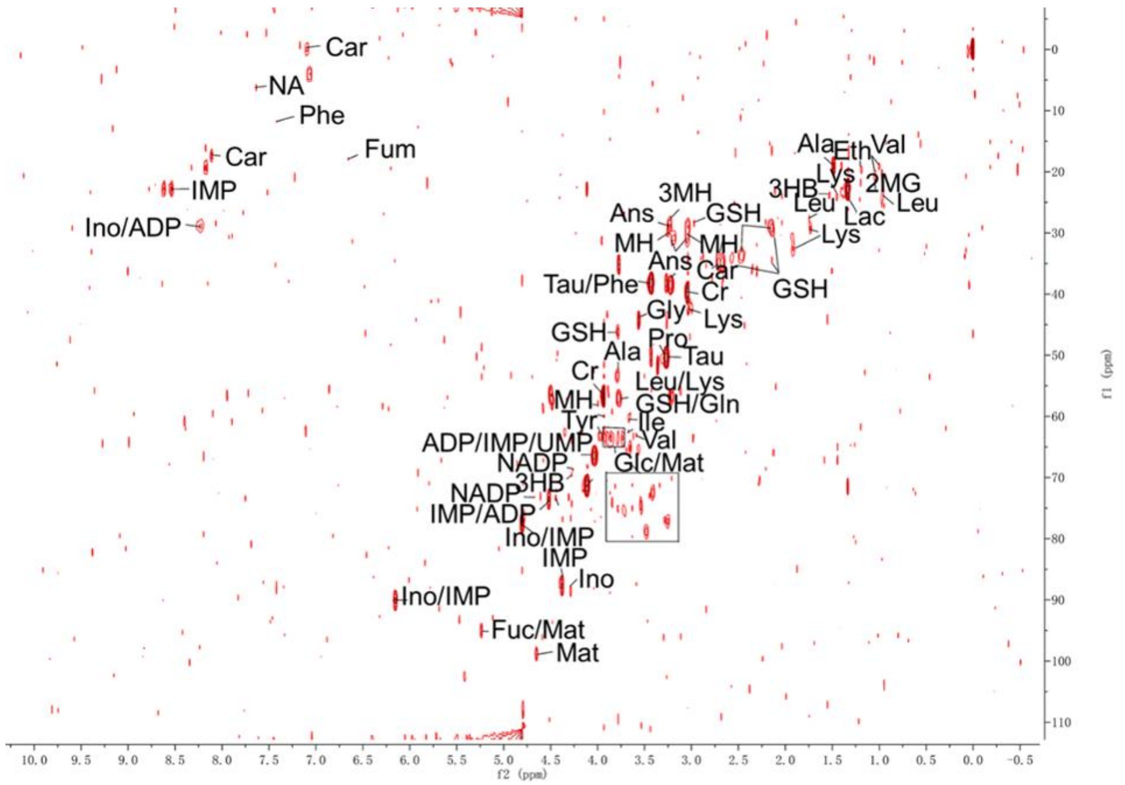

Figure S4. Representative 2D ^{1}H-^{13}C HSQC spectrum recorded on aqueous extracts from the three groups of mouse gastrocnemius. The NMR experiments were performed at 25 °C with a Bruker Avance III 850 MHz spectrometer.

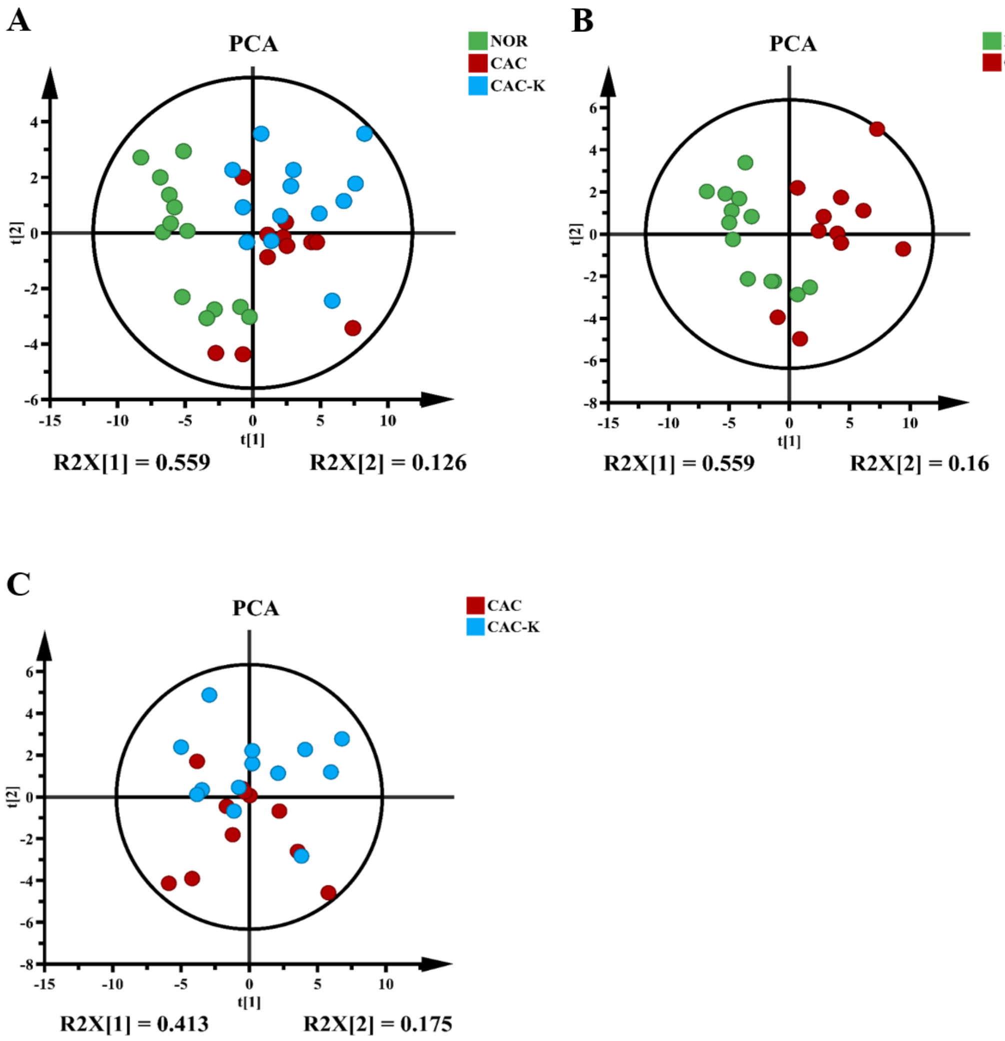

Figure S5. Pattern recognition analysis for the 1D ^{1}H-NMR spectra recorded on aqueous metabolites extracted from the three groups of mouse gastrocnemius. (A-C) PCA scores plots for the three groups (A), the CAC and NOR groups (B), the CAC-K and CAC groups (C).

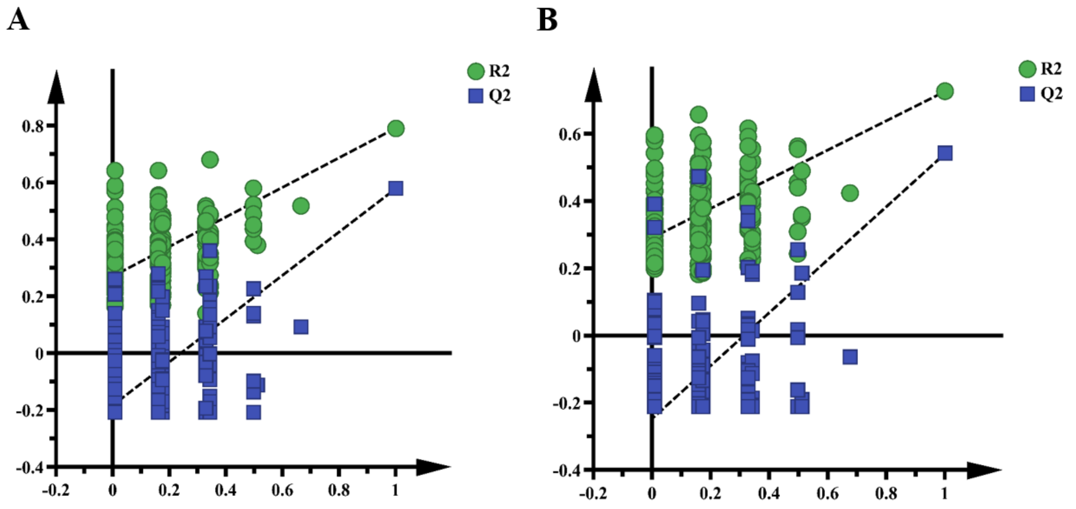

Figure S6. Cross-validation plots of the PLS-DA models for CAC *vs.* NOR and CAC-K *vs.* CAC. (A) CAC *vs.* NOR, (B) CAC-K *vs.* CAC. Response permutation tests were performed to verify the robustness of the established PLS-DA models (200 cycles). The green circle is R2 (cum) standing for the explained variance of the model. The blue square is Q2 (cum) denoting the predictive ability of the model.

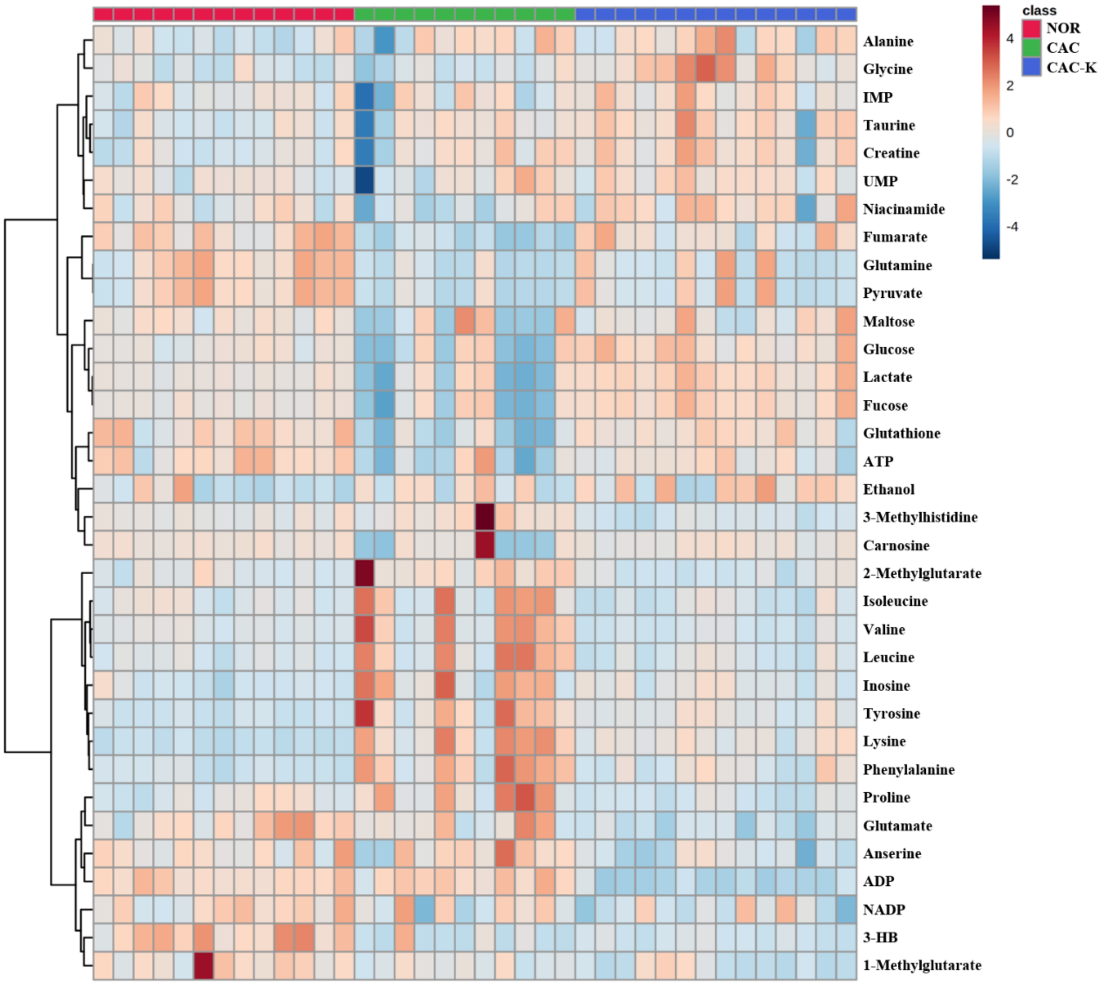

Figure S7. Heat map plot of relative levels of the identified metabolites in the three groups of mouse gastrocnemius.

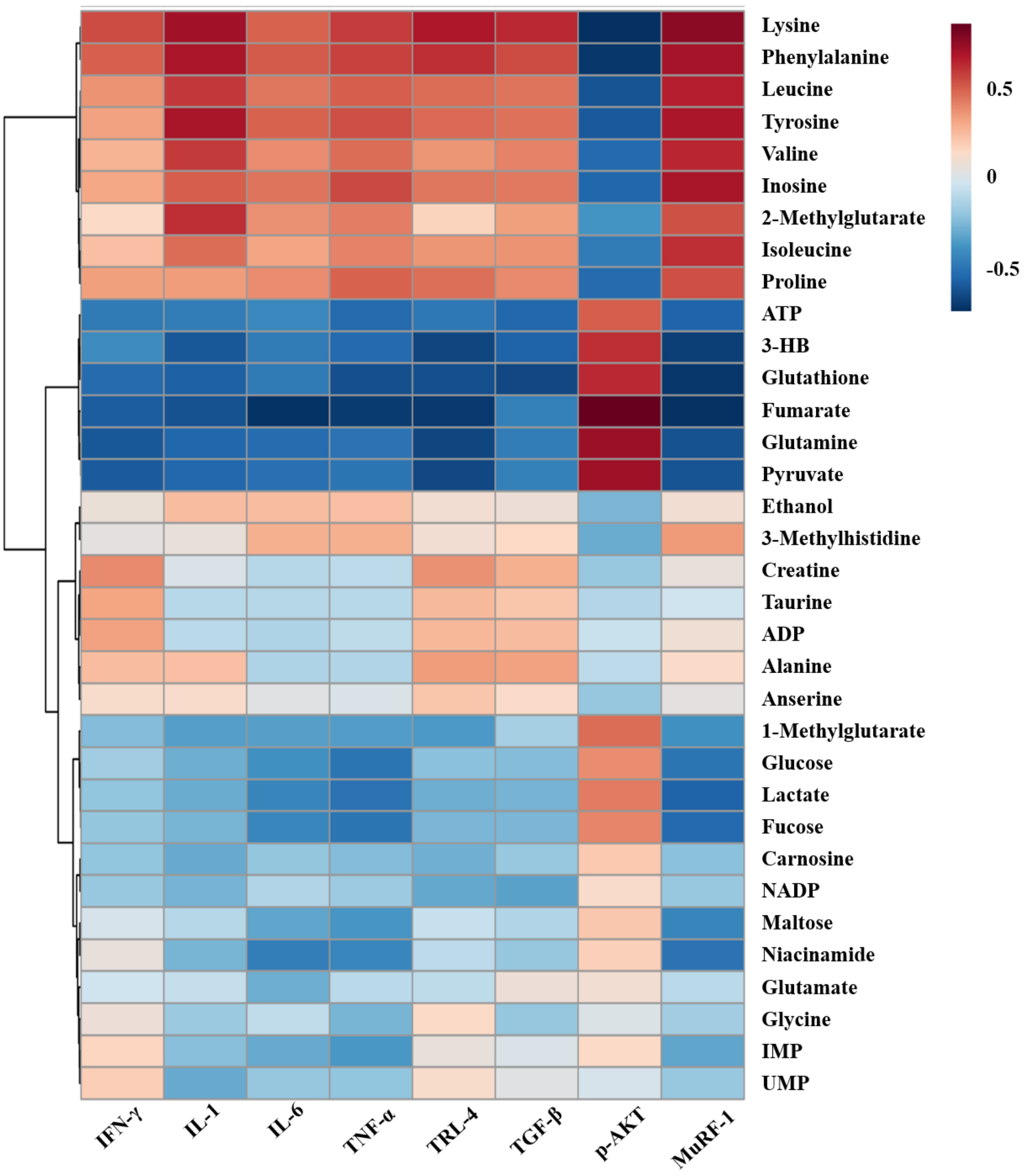

Figure S8. Heat map plots of Pearson's correlations of intergroup changes in serum levels of inflammatory factors, expressions of catabolic and anabolic proteins with those in relative levels of the identified gastrocnemius metabolites for the analysis of CAC *vs.* NOR.

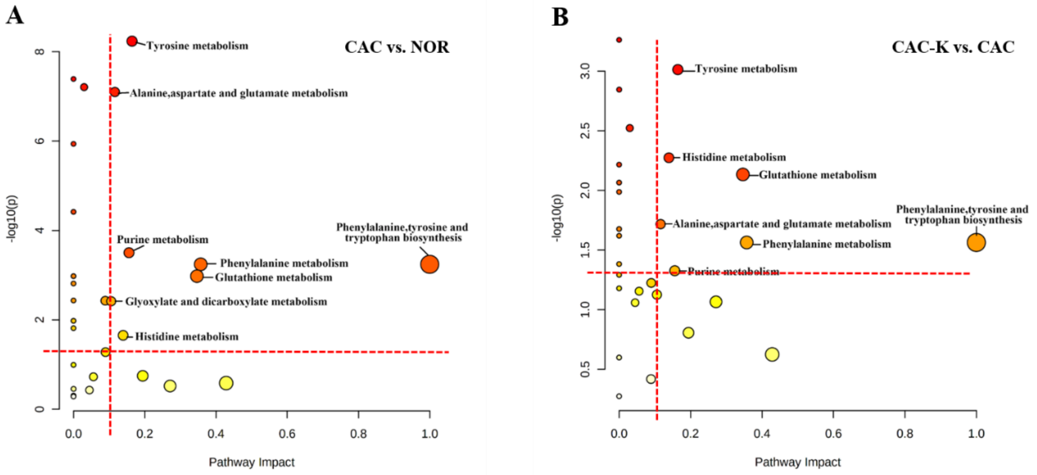

Figure S9. Metabolic pathways analyses for the pairwise comparisons of CAC vs. NOR and CAC-K vs. CAC based on relative levels of the identified metabolites. (A, B) Significantly altered metabolic pathways in the CAC group relative to the NOR group (A), and in the CAC-K group relative to the CAC group (B).
