# Supplementary figures and images for: Exploring the Therapeutic Potential of Ethyl 3-Hydroxybutyrate in Alleviating Skeletal Muscle Wasting in Cancer Cachexia
Source: Biomolecules. 2023 Aug 30;13(9):1330. doi: 10.3390/biom13091330 (PMC10527383; doi:10.3390/biom13091330)

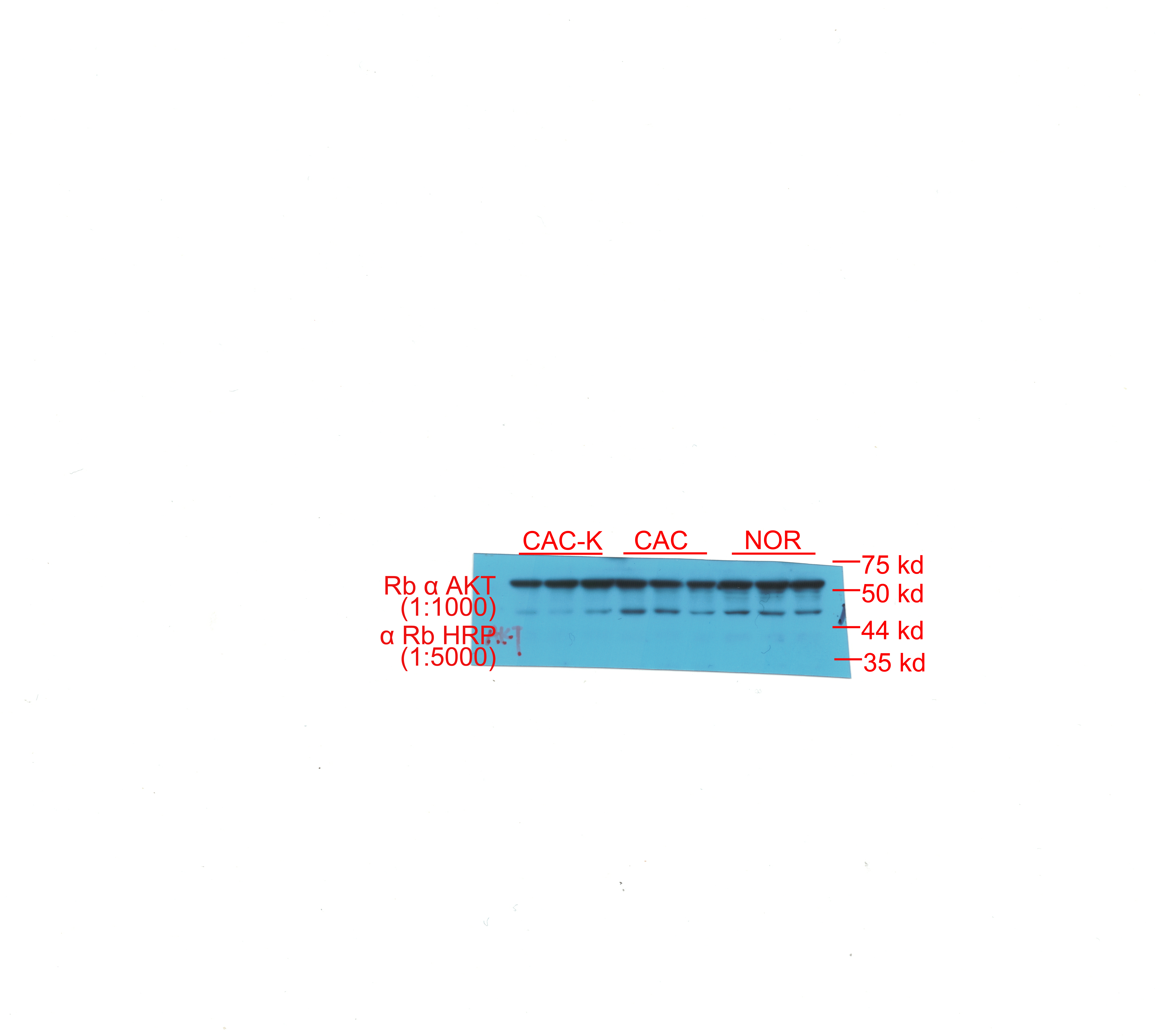

Supplement: Supplementary file 1 [file biomolecules-13-01330-s001.zip › biomolecules-2551713-original images/akt-1230.tif]

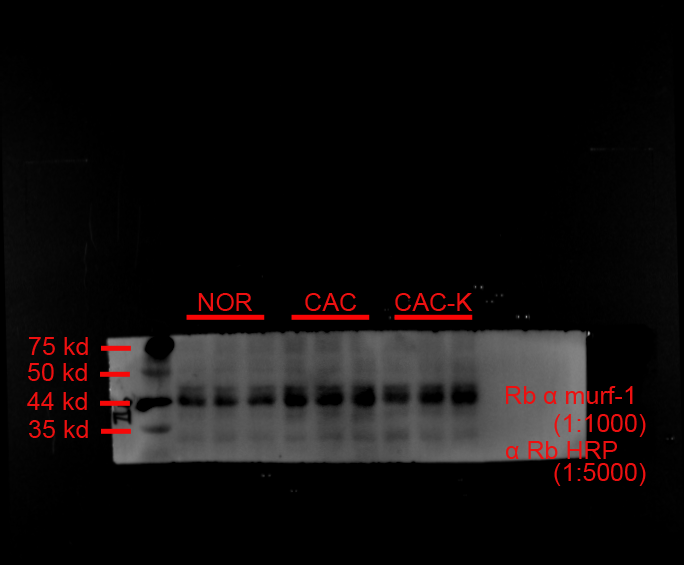

Supplement: Supplementary file 1 [file biomolecules-13-01330-s001.zip › biomolecules-2551713-original images/murf1-1230.tif]
